# Two open-label, single arm, non-randomized phase II studies of irinotecan for the treatment of metastatic breast cancer in patients with increased copy number of the topoisomerase I gene

**DOI:** 10.1186/s12885-019-5788-9

**Published:** 2019-06-13

**Authors:** Iben Kümler, Eva Balslev, Jan Stenvang, Nils Brünner, Bent Ejlertsen, Erik Hugger Jakobsen, Dorte Lisbet Nielsen

**Affiliations:** 1 Department of Oncology, Herlev and Gentofte Hospital, Herlev Ringvej 75, DK-2730 Herlev, Denmark; 2 Department of Pathology, Herlev and Gentofte Hospital, Herlev Ringvej 75, DK-2730 Herlev, Denmark; 3 Institut for Lægemiddeldesign og Farmakologi, Jagtvej 160, 2100 København Ø, Denmark; 4 Department of Oncology, Rigshospitalet, Blegdamsvej 9, DK-2100 Copenhagen, Denmark; 5 Department of Oncology, Vejle Sygehus, Beriderbakken 4, DK-7100 Vejle, Denmark

## Abstract

**Background:**

Treatment options in metastatic breast cancer are limited. New therapies preferable with predictive biomarkers are needed. The aim of these trials was to investigate if gene copy number of the topoisomerase 1 gene was predictive of response to the topoisomerase inhibitor irinotecan.

**Methods:**

Two open-label, single-arm phase II studies including HER2 positive and negative patients were conducted. Patients were eligible for inclusion if the primary tumor or a metastatic lesion had increased expression of the topoisomerase 1 gene defined as a TOP1 gene copy number of ≥4 or a TOP1/CEN20 ratio of ≥2. Patients were treated with irinotecan +/− trastuzumab weekly for 4 weeks following 2 weeks break, until progression or unacceptable toxicities. Evaluation scans were performed every 6 weeks. Primary endpoint was clinical benefit rate defined as the fraction of patients with stable disease for ≥4 months.

**Results:**

The pre-planned number of 18 patients in each trial was not reached, thus no formal statistical analysis could be performed. Nine patients with HER2 negative disease and three patients with HER2 positive disease were included. Three patients obtained a partial remission and two patients had SD.

**Conclusions:**

The trials did not include the planned number of patients. No association between gene copy number of the topoisomerase 1 gene and response to irinotecan could be proved, however a clinical benefit was found in 5/12 patients and in 2/3 patients with HER2 positive disease. This could call for further investigation of the drug in the metastatic setting, especially in HER2 positive BC.

**Trial registration:**

Eudract registration numbers 2012–002348-26 and 2012–002347-23. Registration date August 20th 2012.

## Introduction

Despite improvements in the adjuvant treatment of breast cancer (BC), still about 20% of patients with primary BC will experience loco-regional or distant recurrence [1]. Chemotherapy is the only established option for patients with oestrogen receptor (ER) negative and human epidermal receptor 2 (HER2) negative disease. In HER2 positive disease, the blocking of HER2 signalling is essential and is to a great extent combined with chemotherapy. In patients with ER positive, advanced disease chemotherapy is also recommended in case of rapid progression or suspicion of endocrine resistance. Sequential monotherapy with a cytotoxic drug is recommended by international guidelines in any case of advanced disease. Anthracyclines or taxanes are recommended as first-line chemotherapy for those patients who have not received the drugs in the adjuvant setting. In the western world, a large number of women have been exposed to both drugs at the time of recurrence, excluding their use in the metastatic setting. Other treatment options consist of a number of standard drugs used in random order, with varying response rates, often declining with increasing numbers of treatment lines [2–5]. None of the chemotherapeutics used today are known to be associated with any biomarkers predictive of response.

Irinotecan is a topoisomerase I (TOP1) inhibitor widely used in the treatment of colorectal cancers but only investigated for the use in metastatic BC in a very limited number of studies [6]. Clinical trials have shown modest response rates in metastatic BC, ranging from 5 to 23% in unselected populations, often including patients with several prior treatment regimens for metastatic disease [6].

Tumor levels of *TOP1* have been proposed as a potential biomarker for response to irinotecan. Both gene copy number (CN), *TOP1*/centromere 20 (CEN-20) ratio, protein expression and mRNA expression have been used to identify expression levels of *TOP1* in colorectal cancer but with conflicting outcome [7–11].

Previously, we have shown that approximately 30% of patients with breast cancer are amplified for the *TOP1* gene [12].

Thus, we decided to investigate the efficacy of irinotecan for treatment of patients with metastatic BC and increased CN of the *TOP1* gene.

## Materials and methods

### Study design and objectives

Two identical trials including HER2 positive respectively HER2 negative patients were conducted. Both trials were open label, single-arm, non-randomized, multi-center, phase II studies. Based on Simon’s two-stage Minimax design, using a level of significance of 0.05 (α = 0.05) and a power of 80% (β = 0.20), 19 patients were planned to be included in each of the trials in order to find a clinical benefit rate (CBR) of at least 30%. If less than 7/19 patients obtained clinical benefit (CB), further inclusion would be ceased. If 7 or more patients obtained clinical benefit, another 20 patients would be included.Primary endpoint was CBR defined as the fraction of patients obtaining stable disease for ≥4 months, complete or partial response according to RECIST criteria version 1.1. Secondary endpoints included time to progression, time to death and toxicity.

Both trials were multi center, including 7 Danish departments of Oncology and endorsed and organized in collaboration with the Danish Breast Cancer Cooperative Group.

### Patients

Eligibility criteria included progressive disease, a maximum of 4 previous chemotherapy regimens for metastatic disease, measurable disease by RECIST 1.1 [13], and increased CN of the *TOP1* gene.

Any previous endocrine therapy was allowed.

Both studies were approved by the local Ethics Committee and the Danish Medicines Agency (H-1-2012-066 and H-1-2012-065, Eudract 2012–002348-26 and 2012–002347-23).

### TOP1 analysis

*TOP1* gene CN was determined using FISH (fluorescence in situ hybridization) as previously described [12]. Patients were considered eligible if *TOP1* gene copy number CN in either the primary tumour or metastatic lesion was ≥4 and/or the *TOP1*/CEN20 ratio was ≥2.

### Treatment

Patients with HER2 negative disease were initially treated with intravenous irinotecan 100 mg/m^2^ weekly for 4 weeks followed by two weeks break. The dose was subsequently reduced to 75 mg/m^2^ weekly due to toxicities. Based on a review of previous clinical trials with irinotecan in metastatic BC the initial dose of 100 mg/m^2^ was chosen as this regime had produced the best response rates [6, 14].

For patients with HER2 positive disease the same treatment was administered adding trastuzumab 6 mg/kg every third week. Treatment was continued until disease progression or unacceptable toxicity.

### Evaluation

CT scans were performed before treatment initiation and then every 6 weeks and assessed according to RECIST version 1.1.

## Results

From October 2012 to July 2016, 740 biopsies were collected for *TOP1* analyses. 46 samples were not suitable for analysis due to either poor quality of the FISH signals or lack of tumor tissue in the sample. Thus 694 biopsies were eligible for analysis. 477 samples (69%) were primary mamma carcinomas, the remaining were obtained from various metastatic lesions. The *TOP1/*CEN ratio was ≥2 in 7% of the samples. The ratio varied from 0,35 to 8,18 with a median ratio of 1,33 (Table 1).

**Table 1:** TOP1 analyses

No of Analyses	694
No of patients	580
No of patients with more than 1 biopsy	109
TOP1/CEN20 (median)	0,35–8,18 (1,33)
Gene copy number (median)	1,01–15 (2,63)

A *TOP1* gene CN of ≥4 was found in 15.6% of the samples ranging from 1,01 to 15 and with a median of 2,63 CN. An increase in both *TOP1/*CEN ratio and *TOP1* gene CN was found in 5% of the samples. Totally 18% of the samples exhibited an increased *TOP1* expression either in the *TOP1*/CEN 20 ratio, the gene CN or both.

Nine patients were included in the HER2 negative protocol while three patients were included in the HER2 positive protocol.

The reason for including only 12 patients in total was mainly clinician’s reluctance to try an investigational drug prior to standard therapies. As up to 4 lines of previous chemotherapies were allowed, several eligible patients were never offered treatment in the protocol as their performance status had deteriorated once standard treatments were completed.

Due to poor recruitment the protocols were terminated without fulfilling the planned inclusion number of 19 patients in each of the two protocols. The small number of patients precludes any formal statistical analyses and thus data are only descriptive.

Patient characteristics are given in Table 2. All patients had received at least 2 prior chemo regimens for metastatic disease and 10 patients had received 3 or 4 prior regimens. One patient had primary disseminated disease. All patients were performance status 0–1.

**Table 2:** Patients characteristics

Number of patients	12
Age	Median
50–74	64
PS	
0	4 (33%)
1	8 (67%)
Receptor status	
ER+	12 (100%)
HER2+	3 (25%)
Adjuvant therapy	
chemotherapy	7 (58%)
endocrine	7 (58%)
Primary disseminated disease	1 (8%)
Prior chemo regimens for metastatic disease	
2	2 (17%)
3	6 (50%)
4	4 (33%)
Metastatic sites	
Lymph nodes	5 (42%)
Bone	10 (83%)
Lung	6 (50%)
Pleura	5 (42%)
Liver	8 (67%)
Skin	1 (8%)
Ipsilateral breast	2 (17%)

In the HER2 negative cohort, one patient obtained a partial remission lasting 10 months; two patients had stable disease lasting 8 months and 7 months, respectively. All patients had bone metastases, two had liver metastases and the last patient had lymph node, lung and pleura metastases. The patient with PR had received 3 chemotherapy regimens prior to inclusion. The patients with SD had received 3 and 2 chemotherapy regimens respectively, prior to inclusion.

One patient withdrew after first treatment due to severe comorbidities and three patients withdrew before evaluation was performed due to toxicity (diarrhea). Three patients had progressive disease before 4 months of treatment.

Three patients were enrolled in the HER2 positive study. One patient had partial remission lasting for 10 months and one patient withdrew due to clinical progression prior to evaluation. The third patient withdrew informed consent after 2 treatment cycles, the following CT scan showed partial remission. The patient with stable disease had metastases to lymph nodes and pleura and had received 4 chemo regimens prior to inclusion in the study while the patient who withdrew consent had bone, lung and pleural metastases and had received 3 prior chemotherapy regimens.

Among the 3 patients with PR and SD in the HER2 negative trial two had a mean CN of > 6 while all non-responders had CN below. Looking at the *TOP1*/CEN-20 ratio 2/3 responders had ratios > 2 while only one of the non-responders also exhibited a ratio above 2. The single person with confirmed response in the HER2 positive trial had a *TOP1*/CEN2 ratio of 2.87 and a mean CN of 5 while the patient with PR who withdrew content had a CN of 7 and a *TOP1*/CEN2 of 3.3.

### Toxicities

Initially patients were treated with irinotecan 100 mg/m^2^. However as two out of four patients experienced nausea and diarrhea and were withdrawn from the study, the dose was reduced to 75 mg/m^2^ and hereafter no dose limiting toxicities were observed. Generally the treatment was well tolerated hereafter.

No suspected unexpected serious adverse advents occurred.

## Discussion

The aim of the two studies was to investigate if increased CN of the *TOP1* gene could be used as a predictive biomarker for response to irinotecan. As the planned number of patients was not fulfilled no formal statistical analyses could be done.

Several explanations for the slow recruitment may be found. Prior to initiating the clinical studies we had investigated the frequency of *TOP1* CN gain in normal breast tissue and in samples from primary BC. Based on these results we had anticipated that up to 30% of patients with BC would have increased CN of the *TOP1* gene [15]. In the clinical trials only 19% of patients fulfilled the criteria for increased CN. Furthermore, reluctance to try a new drug before having tried standard drugs lead to a protocol amendment allowing up to 4 prior lines of chemotherapy. This resulted in a substantial number of patients with poor performance status once inclusion into the trials was relevant.

Moreover, specific for the HER2 positive protocol, trastuzumab emtansine (TDM-1) was approved in November 2013 and has proven to be a very effective drug leading to long progression free periods in a large part of patients with HER2 positive disease and thereby postponing the need for experimental treatments.

The chosen cut-off value for *TOP1* CN could be debated. As no clinical data pertaining to *TOP1* in BC have been identified we had to base the cutoff values purely on theoretical assumptions. Dual FISH probes rely on the ratio between the number of signals from the target gene and the number of signals from a reference region on the same chromosome. Thus, a *TOP1*/CEN-20 ratio ≠ 1 would imply an imbalance due to excess copies or loss of either the *TOP1* gene or chromosome 20. However, whether the cutoff should be set at 1.5, 2.0 or even higher is speculative in the absence of clinical data to support such a decision. For the mean CN we chose ≥4 as the threshold for “gain”. This is not totally in line with recommendations for HER2 as these states that in case of a *HER2*/CEN-17 ratio below 2.0 the CN must be ≥6 [16]. As no clinical data could guide us we decided to use ≥4 as this would reflect an excess of the gene even in the case of counting signals in a cell in the S phase. The very limited data from our clinical trials, makes it impossible to investigate if changes in the cutoff value for CN will have any clinical implications although 3/5 responders did have a mean CN of > 6 whereas all non-responders had CN below.

Few studies have reported the use of irinotecan in metastatic BC. The most recent study by Perez et al. included 852 patients randomly assigned to either etrinotecan or treatment of physician’s choice [17].

Patients were heavily pretreated with up to five previous regimens for metastatic disease. The trial did not demonstrate improved overall survival in the etirinotecan group; however the toxicity profile was more favorable for etirinotecan compared to physician’s choice. In this trial several biomarkers were analyzed, among these topoisomerase 1 and 2. So far no results on these biomarkers have been published. To our knowledge, no other studies have investigated the topoisomerases in relation to irinotecan in BC although this might be a biomarker for response if properly investigated and validated.

Furthermore topoisomerase 1 inhibitors like irinotecan might actually be superior to other types of chemotherapy in recurrent BC since a previous study in irinotecan resistant breast cancer cell lines found no cross resistance between taxanes and irinotecan [18] .

However, as our study did not fulfill the planned inclusion no firm conclusions on the presented data can be drawn.

## Conclusion

In conclusion our trials did not succeed in proving an association between gene CN of the *TOP1* gene and response to irinotecan. Since the studies were initiated novel treatment opportunities have emerged in the metastatic setting but even so the need for predictive biomarkers is still desperately wanted. Even though among heavily pretreated patients, 5 out of 12 patients totally and 2 out of 3 patients with HER2 positive disease did have a clinical benefit of treatment with irinotecan, our study did not fulfill the planned inclusion and therefore no firm conclusions can be drawn. Future randomized trials are warranted to identify the best chemotherapeutic options for metastatic breast cancer patients with increased TOP1 gene copy numbers.

## Data Availability

The datasets used during the current study are available from the corresponding author on reasonable request.

## References

[1] Dieci MV (2013). Quantification of residual risk of relapse in breast cancer patients optimally treated. Breast.

[2] Cardoso F (2014). ESO-ESMO 2nd international consensus guidelines for advanced breast cancer (ABC2). Breast.

[3] Rivera E (2010). Management of metastatic breast cancer: monotherapy options for patients resistant to anthracyclines and taxanes. Am J Clin Oncol.

[4] Roche H, Vahdat LT (2011). Treatment of metastatic breast cancer: second line and beyond. Ann Oncol.

[5] Cobleigh MA (2011). Other options in the treatment of advanced breast cancer. Semin Oncol.

[6] Kumler I (2013). A systematic review on topoisomerase 1 inhibition in the treatment of metastatic breast cancer. Breast Cancer Res Treat.

[7] Paradiso A (2004). Topoisomerase-I, thymidylate synthase primary tumour expression and clinical efficacy of 5-FU/CPT-11 chemotherapy in advanced colorectal cancer patients. Int J Cancer.

[8] Braun MS (2008). Predictive biomarkers of chemotherapy efficacy in colorectal cancer: results from the UK MRC FOCUS trial. J Clin Oncol.

[9] Horisberger K (2009). Topoisomerase I expression correlates to response to neoadjuvant irinotecan-based chemoradiation in rectal cancer. Anti-Cancer Drugs.

[10] Vallbohmer D (2006). Molecular determinants of irinotecan efficacy. Int J Cancer.

[11] Nygard SB (2014). Assessment of the topoisomerase I gene copy number as a predictive biomarker of objective response to irinotecan in metastatic colorectal cancer. Scand J Gastroenterol.

[12] Anders C (2014). TBCRC 018: phase II study of iniparib in combination with irinotecan to treat progressive triple negative breast cancer brain metastases. Breast Cancer Res Treat.

[13] Eisenhauer EA (2009). New response evaluation criteria in solid tumours: revised RECIST guideline (version 1.1). Eur J Cancer.

[14] Perez EA (2004). Randomized phase II study of two irinotecan schedules for patients with metastatic breast cancer refractory to an anthracycline, a taxane, or both. J Clin Oncol.

[15] Kumler I (2015). Topoisomerase-1 gene copy aberrations are frequent in patients with breast cancer. Int J Cancer.

[16] Wolff AC (2013). Recommendations for human epidermal growth factor receptor 2 testing in breast cancer: American Society of Clinical Oncology/College of American Pathologists clinical practice guideline update. J Clin Oncol.

[17] Perez EA (2015). Etirinotecan pegol (NKTR-102) versus treatment of physician's choice in women with advanced breast cancer previously treated with an anthracycline, a taxane, and capecitabine (BEACON): a randomised, open-label, multicentre, phase 3 trial. Lancet Oncol.

[18] Jandu H (2016). Molecular characterization of irinotecan (SN-38) resistant human breast cancer cell lines. BMC Cancer.

